# Reply: the ups and downs of passive leg raising

**DOI:** 10.1186/s40635-025-00718-y

**Published:** 2025-01-24

**Authors:** Rogério da Hora Passos, Fernanda Oliveira Coelho, Bruno Zawadzki

**Affiliations:** 1 Departamento de Pacientes Graves—Hospital Israelita Albert Einstein, Av Albert Einstein, 627/701, Morumbi, São Paulo, SP 05652-900 Brazil; 2 Davita Tratamento Renal, Rio de Janeiro, Brazil

## Correspondence

We appreciate the insightful feedback from Dr. Ruste, Dr. Fellahi, and Dr. Jacquet-Lagrèze [1], which has strengthened our understanding of the clinical potential of passive leg raising (PLR) as a predictor of intradialytic hypotension (IDH). Their comments have helped refine the study's focus on practical applications and future directions.

Our study aimed to evaluate PLR and dynamic arterial elastance (Eadyn) as predictors of IDH during hemodialysis with net ultrafiltration. IDH is a frequent complication in dialysis, occurring in up to 30% of sessions and associated with significant morbidity and mortality. Identifying patients at risk for IDH early can improve clinical outcomes, and PLR, being non-invasive and easy to perform, may complement current monitoring methods. Given prior evidence supporting the utility of PLR in other settings, we hypothesized it could predict hemodynamic instability during dialysis [2].

While our results provide preliminary insights, we acknowledge the study’s exploratory nature. Future research should include diagnostic accuracy measures, such as receiver operating characteristic (ROC) analysis, and establish predictive thresholds to enhance PLR’s clinical application. These steps are necessary for the broader use of PLR in clinical practice [3].

A key concern raised was the relationship between fluid responsiveness, indicated by PLR, and patient outcomes, particularly mortality. Fluid responsiveness has been associated with better outcomes in critically ill patients but its prognostic significance in dialysis remains unclear [4]. PLR may reflect cardiovascular reserve, but its link to long-term outcomes like mortality and cardiovascular events is yet to be fully explored. To address this, we are designing a cohort study to investigate whether fluid responsiveness, as measured by PLR, can predict long-term outcomes, including mortality, hospital stay, and cardiovascular events. This study aims to clarify if PLR could be a broader prognostic tool beyond IDH.

Additionally, PLR may help guide real-time clinical decisions during dialysis by identifying patients at risk for hemodynamic instability. This could enable adjustments to dialysis parameters, such as fluid removal rates and vasoactive drug use, particularly in settings where more invasive techniques are not available. However, for PLR to become routine, further validation and the definition of predictive thresholds are necessary [5].

In conclusion, our study suggests that PLR holds potential for identifying patients at risk for IDH. However, further research is needed to confirm its clinical value, establish predictive thresholds, and evaluate its role in real-time decision-making. Our ongoing cohort study will further explore the relationship between PLR, fluid responsiveness, and long-term patient outcomes, aiming to refine fluid management strategies and improve dialysis patient care.

## Data Availability

Not applicable.
